# Safety and Efficacy of Stand-Alone and Hybrid Thoracoscopic Atrial Fibrillation Ablation

**DOI:** 10.1093/ejcts/ezag161

**Published:** 2026-04-27

**Authors:** Luca Aerts, Michal J Kawczynski, Niels J Verberkmoes, Thomas Van Brakel, Justin G L M Luermans, Samuel Heuts, Eva Verbeek, Ricardo Cocchieri, Sacha P Salzberg, Henri Gruwez, Herbert Gutermann, Laurent Pison, Dmitry Elesin, Alexander Bogachev-Prokophiev, Oleg Shelest, Alexandr Troitskiy, Robert Khabazov, Aleksander Zotov, Bart Maesen

**Affiliations:** Department of Cardiothoracic Surgery, Maastricht University Medical Centre, Maastricht 6202 AZ, The Netherlands; Cardiovascular Research Institute Maastricht (CARIM), Maastricht University, Maastricht 6229 ER, The Netherlands; Department of Cardiothoracic Surgery, Maastricht University Medical Centre, Maastricht 6202 AZ, The Netherlands; Cardiovascular Research Institute Maastricht (CARIM), Maastricht University, Maastricht 6229 ER, The Netherlands; Department of Cardiothoracic Surgery, Catharina Ziekenhuis, Eindhoven 5623 EJ, The Netherlands; Department of Cardiothoracic Surgery, Catharina Ziekenhuis, Eindhoven 5623 EJ, The Netherlands; Cardiovascular Research Institute Maastricht (CARIM), Maastricht University, Maastricht 6229 ER, The Netherlands; Department of Cardiology, Maastricht University Medical Centre, Maastricht 6202 AZ, The Netherlands; Department of Cardiothoracic Surgery, Maastricht University Medical Centre, Maastricht 6202 AZ, The Netherlands; Cardiovascular Research Institute Maastricht (CARIM), Maastricht University, Maastricht 6229 ER, The Netherlands; Department of Cardiothoracic Surgery, Onze Lieve Vrouwe Gasthuis, Amsterdam 1091 AC, The Netherlands; Department of Cardiothoracic Surgery, Onze Lieve Vrouwe Gasthuis, Amsterdam 1091 AC, The Netherlands; Department of Cardiothoracic Surgery, Klinik Hirslanden, 8032 Zürich, Switzerland; Department of Cardiothoracic Surgery, Schön Klinik Vogtareuth, 83569 Vogtareuth, Germany; Department of Cardiology and Cardiac Surgery, Ziekenhuis Oost-Limburg, 3600 Genk, Belgium; Department of Cardiology and Cardiac Surgery, Ziekenhuis Oost-Limburg, 3600 Genk, Belgium; Department of Cardiology and Cardiac Surgery, Ziekenhuis Oost-Limburg, 3600 Genk, Belgium; Department of Cardiology and Cardiothoracic Surgery, Meshalkin National Medical Research Center, Novosibirsk 630055, Russia; Department of Cardiology and Cardiothoracic Surgery, Meshalkin National Medical Research Center, Novosibirsk 630055, Russia; Department of Cardiology and Cardiothoracic Surgery, Federal Research and Clinical Center, Moscow 119435, Russia; Department of Cardiology and Cardiothoracic Surgery, Federal Research and Clinical Center, Moscow 119435, Russia; Department of Cardiology and Cardiothoracic Surgery, Federal Research and Clinical Center, Moscow 119435, Russia; Department of Cardiology and Cardiothoracic Surgery, Federal Research and Clinical Center, Moscow 119435, Russia; Department of Cardiothoracic Surgery, Maastricht University Medical Centre, Maastricht 6202 AZ, The Netherlands; Cardiovascular Research Institute Maastricht (CARIM), Maastricht University, Maastricht 6229 ER, The Netherlands

**Keywords:** thoracoscopic AF ablation, hybrid AF ablation, atrial fibrillation, radiofrequency

## Abstract

**Objectives:**

This study aimed to evaluate the long-term efficacy and safety of isolated and hybrid thoracoscopic atrial fibrillation (AF) ablation using a bipolar irrigated radiofrequency clamp in a multicentre registry.

**Methods:**

A retrospective multicentre registry of patients undergoing AF ablation using the bipolar clamp was conducted over the past 13 years (2010-2023). The primary efficacy outcome was freedom of atrial tachyarrhythmias (ATAs), with and without the use of Class I/III antiarrhythmic drugs (AADs). Antiarrhythmic drug use during follow-up was not uniformly documented across centres. The primary safety outcome was the rate of periprocedural complications.

**Results:**

The cohort of 678 patients consisted of a minority of female patients (17.4%), with most patients having longstanding persistent AF (LSPAF) (66.7%), a mean duration of 61 months of AF duration and 33.3% had undergone prior catheter ablation. Freedom from ATA while allowing Class I/III AAD use was 82.3%, 71.5%, and 52.4% at 1, 3, and 5 years, respectively. Freedom from ATA off Class I/III AAD declined from 71.7% at 1 year to 44.2% at 5 years, underscoring the progressive nature of AF and the need for long-term rhythm strategies. Women presented with a more advanced cardiovascular risk profile than men, including older age (60.3 vs 57.3 years), higher CHA2DS2-VA-scores, and more comorbidities. Despite these differences, there were no significant sex-based differences in long-term ATA freedom. There were no significant unadjusted differences in long-term ATA freedom between paroxysmal AF (PAF) and non-PAF. Major complication rate was low.

**Conclusions:**

Isolated and hybrid thoracoscopic AF ablation using the Gemini Clamp demonstrated favourable outcomes with a low complication rate. However, variability in ATA detection methods among centres may have influenced the primary outcome and should be considered when interpreting long-term efficacy results.

**Clinical trial registry number:**

METC-number 2022-3561.

## Graphical abstract

**Figure:**
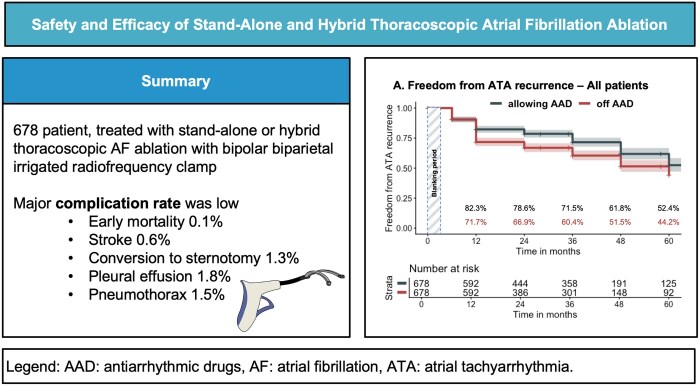

## Introduction

Although catheter ablation is the first-line invasive treatment for atrial fibrillation (AF), its efficacy in persistent and longstanding persistent AF (LSPAF) remains suboptimal.^1^ Isolated and hybrid thoracoscopic AF ablation offer a minimally invasive surgical alternative for the treatment of AF patients. Most contemporary series have reported on the efficacy and safety of thoracoscopic AF ablation, using non-irrigated radiofrequency (RF) devices.^2^ Irrigated RF has the potential to achieve more durable and transmural lesions; however, reports on the efficacy and safety of thoracoscopic AF ablation using irrigated RF clamps are lacking. This international multicentre study focuses on the real-world safety and long-term efficacy of thoracoscopic AF ablation using a bipolar irrigated RF clamp.

## Methods

### Study design

The current study was a multicentre, retrospective registry that aimed to collect procedural and follow-up data on patients who underwent isolated and hybrid thoracoscopic AF ablation using a bipolar irrigated RF clamp (Cardioblate Gemini, Medtronic, MN, USA) during the last 13 year to evaluate the efficacy and safety of this technique. Following local ethical approval, the study was registered at Maastricht University Medical Center (registration number: METC 2022-3561). Ethical approval was obtained from all participating hospitals.

### Study sample

Consecutive patients with paroxysmal AF (PAF), persistent AF, or LSPAF who underwent isolated and thoracoscopic hybrid ablation using a bipolar irrigated RF clamp between 2010 and 2023 were included. Inclusion dates varied by centre, as detailed in **Table S1**. Representing a maximum follow-up of 13 years and a median follow-up duration of 5 years.

### Data collection

Data were extracted from medical records into a standardized electronic case form (ResearchManager). All information was collected from the electronic patient information systems of the Cardiothoracic Surgery and Cardiology departments by study coordinators at each site.

### Procedural aspects

All patients underwent ablation using a bipolar irrigated RF clamp. The clamp was introduced via bilateral thoracoscopic access, allowing precise positioning of the jaws around the target tissue in both the transverse and oblique sinuses. This approach facilitated consistent transmural heating and the creation of 2 continuous U-shaped lesions for complete electrical isolation of the left atrial posterior wall (LAPW). In isolated thoracoscopic procedures, the epicardial lesion set consisted of pulmonary vein isolation (PVI) and LAPW isolation, with left atrial appendage (LAA) closure when feasible. In hybrid procedures, this epicardial lesion set was complemented by a planned endocardial step, allowing additional substrate modification, including cavotricuspid isthmus (CTI) line, mitral isthmus line, and focal ablation when indicated.

In the hybrid group, validation of lesion transmurality was performed using endocardial mapping, whereas in the thoracoscopic group, epicardial pacing with testing of exit block was used for validation. Superior vena cava (SVC) ablation was performed selectively based on electrophysiological considerations, acknowledging the presence of muscular sleeves capable of ectopic activity, and was always achieved epicardially. A simplified schematic of epicardial and endocardial lesion sets is provided in the **Figure S1**.

### Study outcomes

The primary efficacy outcome was long-term freedom from atrial tachyarrhythmia (ATA), both with (allowing antiarrhythmic drug [AAD]) and without the use of AAD (off AAD; composite of ATA recurrence and AAD initiation). Antiarrhythmic drug (AAD) therapy was defined as treatment with Class I or Class III agents; the use of Class II (beta-blockers) or Class IV (calcium-channel blockers) was permitted and not considered AAD failure. This primary outcome was designed to assess the overall efficacy of the surgical technique, specifically ablation with a biparietal bipolar irrigated RF clamp, irrespective of the underlying AF subtype. Therefore, the primary analysis was performed in the overall cohort, with additional stratified analyses for PAF and non-PAF patients reported separately.

The primary end-point was defined either as freedom from ATA or as treatment failure (combination of ATA recurrence or initiation or continuation of Class I or Class III AAD therapy beyond the blanking period). Atrial tachyarrhythmia recurrence after the index hybrid procedure, or the need for any ablation outside the predefined hybrid scheme (including redo catheter ablation), was considered a permanent treatment failure, irrespective of subsequent sinus rhythm (SR) restoration.

The secondary efficacy outcome focused on both unadjusted and adjusted freedom from ATA, allowing and off Class I/III AADs, in patients with PAF and non-PAF history. The primary safety outcome was the rate of periprocedural complications (which was reported separately, and as a composite of pneumo- or haemothorax, or pleural effusion requiring drainage, respiratory failure, pneumonia, pericarditis, stroke, pacemaker implantation, phrenic nerve palsy, and/or myocardial infarction). Additionally, the influence of sex differences on long-term freedom from ATA was assessed, considering cardiovascular profiles and procedural variables.

Long-term data regarding oral anticoagulation use, stroke, and major bleeding events were not uniformly collected or adjudicated across centres, as the registry primarily focused on procedural and rhythm-related outcomes.

### Follow-up

Rhythm monitoring was conducted at 1, 2-, 3-, 4-, and 5-years post-procedure. Follow-up assessments included electrocardiogram (ECG), 24-hour, 48-hour, 72-hour, or 7-day Holter monitoring, and, where applicable, Implantable Loop Recorder (ILR) data (**Table S2**). In line with current guidelines, treatment success was defined as the absence of any ATA lasting more than 30 seconds.^1^ Data on AAD therapy were collected; however, detailed information regarding exact duration of treatment, temporary amiodarone use, and timing of discontinuation was not consistently available across all participating centres. No standardized protocol for AAD discontinuation was applied.

### Statistical analysis

All variables were assessed for missing values, and multiple imputation was performed when missing data were present. Multiple imputation was conducted in SPSS (version 31.0) using the default fully conditional specification (FCS) approach. Categorical variables were imputed using SPSS’s default multinomial logistic regression method within the FCS framework, and continuous variables were imputed using linear regression-based methods. We generated 5 imputed datasets, performed all analyses across imputations, and pooled the estimates using Rubin’s Rules.

All continuous variables were evaluated for data distribution using the Shapiro-Wilk test before any analyses were conducted. Continuous variables were presented as means with corresponding standard deviations (SDs) or as medians with interquartile ranges (IQRs), depending on the type of distribution. Categorical variables were presented as absolute values with proportions (%). Differences between rhythm groups were analysed using Student’s t-test or Mann-Whitney U tests for continuous data, depending on the data distribution and homogeneity of variance. Group differences for categorical variables were assessed with the Chi-squared test.

Unadjusted associations with long-term freedom from ATA, with and without the use of Class I/III AADs, were assessed using Cox proportional hazards models. Long-term freedom from ATA was presented in Kaplan-Meier (KM) curves based on the results from the unadjusted Cox proportional hazards models. Based on unadjusted results, along with predictors previously described in the literature, such as age and sex, adjusted mixed-effects Cox frailty models—further adjusted for frailty (study centre effect)—were created to identify clinical variables associated with long-term freedom from ATA. The proportional hazards assumption was assessed visually and tested using Schoenfeld residuals (for which *P* < .05 denoted violation of the proportional hazards assumptions). Sensitivity analyses were conducted by excluding patients with incomplete follow-up and using alternatives definitions of ATA recurrence.

All *P*-values were based on 2-sided statistical tests, with a threshold of <.05 to determine statistical significance. All statistical analyses were performed using RStudio (Version 1.2.1335; packages: “survival,” “survminer,” “coxme”) and SPSS software (Version 31.0, IBM Corp., Armonk, NY).

## Results

### Overall patient characteristics

A total of 678 patients from 8 centres were included, with 613 (90.4%) patients undergoing isolated and 65 (9.6%) patients undergoing hybrid thoracoscopic AF ablation. Baseline characteristics are presented in **Table 1**. The mean age was 57.8 (SD: 8.7) years, 17.4% were female, and most patients had LSPAF (66.7%). The median duration of AF history was 45 (IQR: 24-84) months, with 33.3% prior catheter ablations. The median left atrial volume index (LAVI) was 55.36 (SD: 16.44) mL/m^2^.

**Table 1.** Baseline Patient Characteristics

Variable	Overall cohort (*n* = 678)	PAF (*n* = 124)	Non-PAF (*n* = 554)	*P*-value
*Patient characteristics*				
Preoperative rhythm history (%)	678 (100)	124 (100)	554 (100)	*NA*
Paroxysmal AF (%)	124 (18.3)	124 (100)	0 (0)	
Persistent AF (%)	102 (15.0)	0 (0)	102 (18.4)	
Longstanding persistent AF (%)	452 (66.7)	0 (0)	452 (81.6)	
Prior catheter ablation (%)	224 (33.3)	47 (38.2)	177 (32.2)	.204
AF duration, months (IQR)	45 (IQR: 24-84)	42 (IQR: 24-84)	48 (IQR: 21-84)	.346
Age, years (SD)	57.82 (SD: 8.71)	60.09 (SD: 9.01)	57.31 (SD: 8.57)	.001
Female sex (%)	118 (17.4)	36 (29.0)	82 (14.8)	<.001
BSA, m^2^ (SD)	2.15 (SD: 0.20)	2.10 (SD: 0.20)	2.16 (SD: 0.20)	.004
BMI, kg/m^2^ (SD)	29.48 (SD: 4.63)	28.32 (SD: 4.47)	29.73 (SD: 4.63)	.002
Hypertension (%)	470 (69.3)	68 (54.8)	402 (72.6)	<.001
Diabetes mellitus (%)	45 (6.6)	12 (9.7)	33 (6.0)	.132
Peripheral vascular disease (%)	35 (5.2)	5 (4.0)	30 (5.4)	.529
Myocardial infarction (%)	33 (4.9)	4 (3.2)	29 (5.2)	.347
History of PCI (%)	21 (3.1)	5 (4.0)	15 (2.7)	.431
Stroke (%)	48 (7.1)	8 (6.5)	40 (7.2)	.763
Pulmonary embolism (%)	5 (0.7)	1 (0.8)	4 (0.7)	.921
COPD (%)	24 (3.5)	5 (4.0)	19 (3.4)	.743
Congestive heart failure (%)	8 (1.2)	4 (3.2)	4 (0.7)	.020
Kidney dysfunction (%)	10 (1.5)	4 (3.3)	6 (1.1)	.072
Sleep apnoea (%)	22 (3.2)	8 (6.5)	14 (2.5)	.026
Smoking history (%)	258 (38.1)	52 (41.9)	206 (37.2)	.325
CHA₂DS₂-VA-score (IQR)	1 (IQR: 1-2)	1 (IQR: 0-2)	1 (IQR: 1-2)	.799
*Preoperative medication*				
No antiarrhythmic drugs (%)	160 (24.8)	44 (37.3)	116 (22.1)	<.001
Flecainide (%)	52 (8.1)	26 (22.0)	26 (4.9)	<.001
Procainamide (%)	3 (0.5)	0 (0)	3 (0.6)	.411
Sotalol (%)	87 (13.5)	27 (22.9)	60 (11.4)	<.001
Amiodarone (%)	337 (52.3)	20 (16.9)	317 (60.3)	<.001
Other (%)	5 (0.8)	1 (0.8)	4 (0.8)	.924
Beta blocker (%)	225 (33.2)	69 (55.6)	156 (28.2)	<.001
Digitalis (%)	47 (6.9)	7 (5.6)	40 (7.2)	.532
Oral anticoagulation (%)	636 (93.8)	115 (92.7)	521 (94.0)	.586
*Preoperative transthoracic echocardiography*				
LVEF, % (SD)	57.62 (SD: 7.45)	56.13 (SD: 7.39)	57.94 (SD: 7.43)	.057
LAD, mm (SD)	48.57 (SD: 7.68)	44.57 (SD: 6.47)	49.05 (SD: 7.68)	<.001
LAV, mL (SD)	118.47 (SD: 36.46)	81.57 (SD: 28.79)	124.65 (SD: 33.88)	<.001
LAVI, mL/m^2^ (SD)	55.36 (SD: 16.44)	39.47 (SD: 13.59)	58.05 (SD: 15.34)	<.001
Mitral valve regurgitation (%)^a^	79 (11.7)	19 (15.3)	60 (10.8)	.158
Tricuspid valve insufficiency (%)^b^	59 (8.7)	13 (10.5)	46 (8.3)	.436

a Defined as moderate or more severe regurgitation.

b Defined as moderate or more severe regurgitation.

Abbreviations: AF, atrial fibrillation; BMI, body mass index; BSA, body surface area; COPD, chronic obstructive pulmonary disease; LAD, left atrial diameter; LAV, left atrial volume; LAVI, left atrial volume indexed for body surface area; LVEF, left ventricle ejection fraction; PAF, paroxysmal atrial fibrillation; PCI, percutaneous coronary intervention; SD, standard deviation.

### Patient characteristics by rhythm history

Most demographic characteristics did not differ significantly between patients with PAF and those with non-PAF (**Table 1**). However, patients with PAF were older (60 [SD: 9.01] vs 57 [SD: 8.57] years, *P* = .001) and had a higher proportion of females (29% vs 14.8%, *P* < .001). Additionally, patients with non-PAF had a higher body mass index (BMI) (29.73 [SD: 4.63] kg/m^2^ vs 28.32 [SD: 4.74] kg/m^2^, *P* = .002), and a higher prevalence of hypertension (72.6% vs 54.8%, *P* < .001).

### Procedural characteristics

Patients with PAF more frequently underwent hybrid thoracoscopic ablation (19.4% vs 7.4%, *P* < .001) and were more frequently in SR at the start of the procedure (64.2% vs 10.8%, *P* < .001), compared to non-PAF (**Table 2**). Overall, the mean procedure time was 136.7 [SD: 38.4] min, with no significant differences between PAF and non-PAF patients.

**Table 2.** Procedural Characteristics

Variable	Overall cohort (*n* = 678)	PAF (*n* = 124)	Non-PAF (*n* = 554)	*P*-value
Type of surgical ablation (%)	678 (100)	124 (100)	554 (100)	<.001
Thoracoscopic (%)	613 (90.4)	100 (80.6)	513 (92.6)	
Hybrid thoracoscopic (%)	65 (9.6)	24 (19.4)	41 (7.4)	
SR at start of procedure (%)	136 (20.5)	77 (64.2)	59 (10.8)	<.001
SR at end of procedure (%)	634 (94.9)	119 (98.3)	515 (94.1)	.287
ECV at end of procedure (%)	394 (59.0)	40 (33.3)	354 (64.6)	<.001
SVC isolation (%)	67 (12.1)	24 (23.3)	43 (9.6)	<.001
No LAA management (%)	24 (3.6)	11 (9.2)	13 (2.4)	<.001
AtriClip (%)	139 (21.0)	41 (34.2)	98 (18.1)	<.001
Epicardial stapler device (%)	499 (75.4)	68 (56.6)	431 (79.5)	<.001
Procedure time, minutes (SD)	136.71 (SD: 38.43)	143.40 (SD: 48.79)	135.39 (SD: 35.94)	.148

Abbreviations: ECV, electrical cardioversion; LAA, left atrial appendage; PVI, pulmonary vein isolation; SD, standard deviation; SR, sinus rhythm; SVC, superior vena cava.

### Postoperative outcomes

Periprocedural complication are described in **Table 3**. Overall, early mortality (0.1%) and stroke (0.6%) was very low. Conversion to sternotomy occurred in 9 patients (1.3%). Most observed complications were pleural effusion (1.8%) and pneumothorax (1.5%) requiring drainage. The majority of patients remained in SR during hospitalization (88.6%).

**Table 3.** Postoperative Course and Early Outcomes

Variable	Overall cohort (*n* = 678)	PAF (*n *= 124)	Non-PAF (*n* = 554)	*P*-value
Hospital admission, days (IQR)	7 (4-9)	4 (3-6)	7 (5-9)	<.001
Rhythm during admission (%)	678 (100)	124 (100)	554 (100)	.440
Sinus rhythm (%)	601 (88.6)	107 (86.3)	494 (89.2)	.361
AF (%)	49 (7.2)	11 (8.9)	38 (6.9)	.434
Atrial flutter (%)	9 (1.3)	1 (0.8)	8 (1.4)	.575
Atrial tachycardia (%)	3 (0.4)	0 (0)	3 (0.5)	.412
Junctional rhythm (%)	10 (1.4)	4 (3.2)	6 (1.1)	.074
Other (%)	6 (0.9)	1 (0.8)	5 (0.9)	.916
In-hospital mortality (%)	1 (0.1)	0 (0)	1 (0.2)	.636
Conversion to sternotomy (%)	9 (1.3)	3 (2.4)	6 (1.1)	.240
Postoperative complications (%)	60 (8.8)	19 (15.3)	41 (7.4)	.005
Pneumothorax requiring drainage (%)	10 (1.5)	3 (2.4)	7 (1.3)	.334
Pleural effusion requiring drainage (%)	12 (1.8)	0 (0)	12 (2.2)	.098
Haemothorax requiring drainage (%)	3 (0.4)	1 (0.8)	2 (0.4)	.499
Respiratory failure (%)	7 (1.0)	2 (1.6)	5 (0.9)	.479
Pneumonia (%)	6 (0.9)	4 (3.2)	2 (0.4)	.002
Pericarditis (%)	7 (1.0)	5 (4.0)	2 (0.4)	<.001
Stroke (%)	4 (0.6)	0 (0)	4 (0.7)	.342
Permanent PM implantation (%)	7 (1.0)	2 (1.6)	5 (0.9)	.479
Phrenic nerve palsy (%)	1 (0.1)	0 (0)	1 (0.2)	.636
Myocardial infarction (%)	2 (0.3)	1 (0.8)	1 (0.2)	.245

Abbreviations: AF, atrial fibrillation; ICU, intensive care unit; IQR, interquartile range; PM, pacemaker.

Five patients were lost to follow-up, and 2 patients died from unknown causes. After 1 year of follow-up, 60.5% of the patients were using oral anticoagulation.

### Unadjusted freedom from ATA

**Figure 1.**
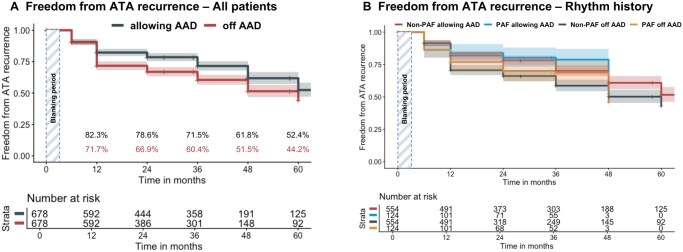
Kaplan-Meier curves for freedom from ATA recurrence allowing Class I/III AAD vs off Class I/III AAD for (A) all patients; (B) paroxysmal AF vs non-paroxysmal AF patients (Rhythm history). Abbreviations: AAD, antiarrhythmic drugs; ATA, atrial tachyarrhythmia; PAF, paroxysmal atrial fibrillation.

**Figure 2.**
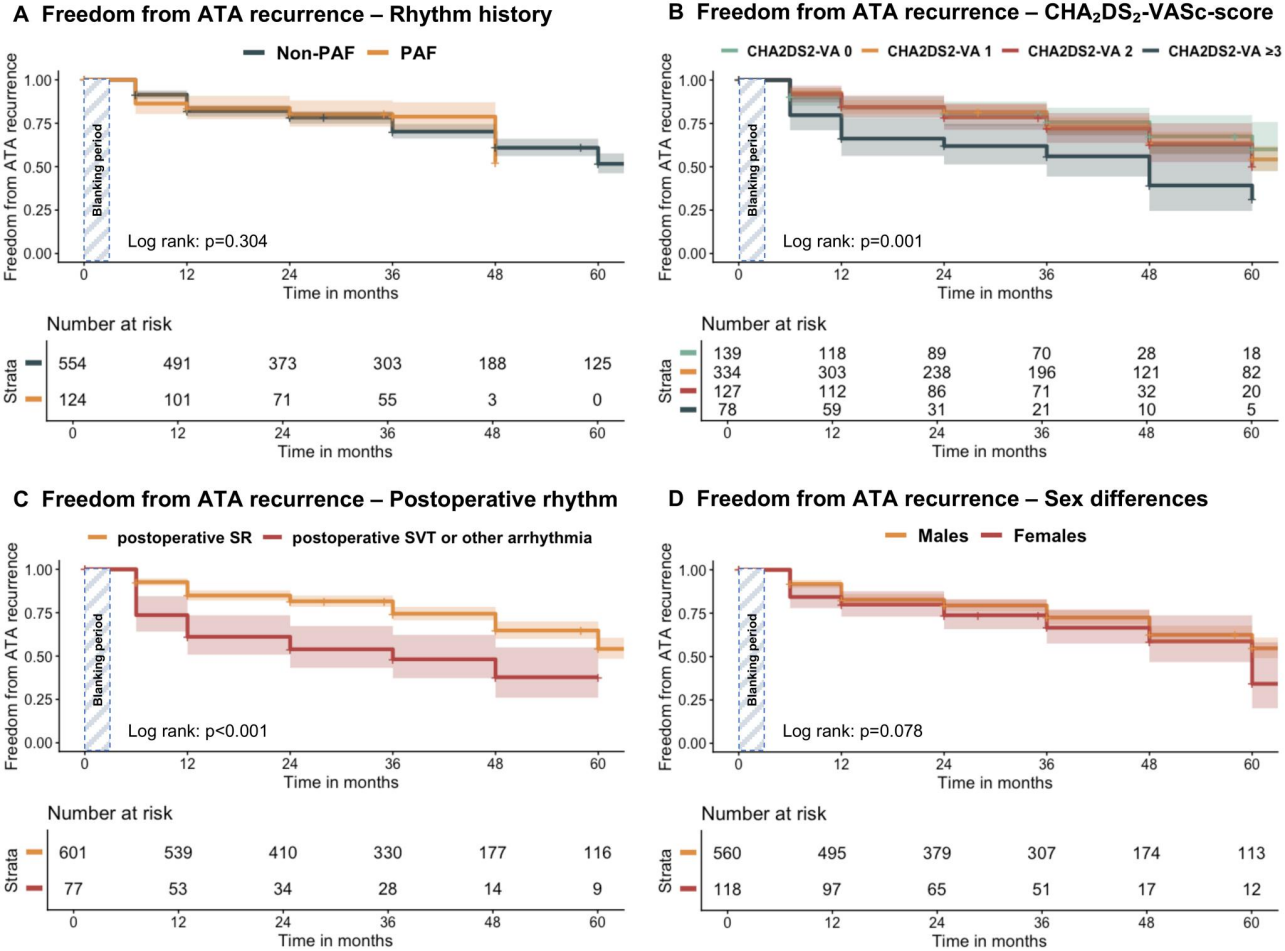
Kaplan-Meier curves for freedom from ATA recurrence for (A) paroxysmal AF vs non-paroxysmal AF patients (Rhythm history); (B) CHA_2_DS_2_-VA-score 0, CHA_2_DS_2_-VA-score 1, CHA2DS2-VA-score 2, CHA_2_DS_2_-VA-score ≥3; (C) Postoperative rhythm: SR vs supraventricular tachycardia or any other arrhythmia; (D) for female vs male patients (sex differences). Abbreviations: ATA, atrial tachyarrhythmia; PAF, paroxysmal atrial fibrillation; SR, sinus rhythm.

Overall freedom from ATA allowing Class I/III AAD use was 82.3%, 71.5%, and 52.4% at 1 year, 3 years, and 5 years of follow-up, respectively (**Table S3** and **Figure 1A**). Freedom from ATA off Class I/III AAD was 71.7%, 60.4%, and 44.2% at 1 year, 3 years, and 5 years of follow-up, respectively (**Figure 1A**). There was no significant difference in freedom from ATA between patients with PAF and non-PAF, both when allowing and off Class I/III AADs (**Figures 1B** and **2A**). Prior catheter ablation (hazard ratio [HR]=1.35, 95% CI: 1.01-1.79, *P* = .043), older age (HR = 1.03, 95% CI: 1.01-1.05, *P* < .001), history of stroke (HR = 1.40, 95% CI: 1.10-1.77, *P* = .006), chronic obstructive pulmonary disease (COPD) (HR = 2.15, 95% CI: 1.17-3.96, *P* = .014), smoking history (HR = 1.36, 95% CI: 1.03-1.81, *P* = .028), and higher preoperative CHA_2_DS_2_-VA-score (HR = 1.27, 95% CI: 1.20-1.34, *P* < .001) were all associated with lower long-term freedom from ATA in the unadjusted analysis (**Table S4**). Analysis of the CHA_2_DS_2_-VA-score stratified by different classes revealed that a score of ≥3 was particularly associated with lower long-term freedom from ATA (**Figure 2B**). Early AF recurrences during hospital admission were significantly associated with lower freedom from ATA (HR = 2.65, 95% CI: 1.77-3.98, *P* < .001) (**Figure 2C**).

A secondary analysis for treatment failure (composite of ATA recurrence or Class I/III AAD initiation) revealed similar preoperative clinical variables, including older age (HR = 1.02, 95% CI: 1.01-1.04, *P* = .003), and higher CHA_2_DS_2_-VA-score (HR = 1.15, 95% CI: 1.09-1.21, *P* < .001) (**Table S5**). Again, early AF recurrences during hospital admission were significantly associated with lower freedom from ATA or Class I/III AAD initiation (HR = 2.35, 95% CI: 1.63-3.41, *P* < .001) (**Table S6**).

### Adjusted predictors for ATA recurrence

Based on the outcomes from the unadjusted analysis, several adjusted mixed-effects Cox models were developed (**Table S6**). The first model included preoperative clinical variables, with separate inclusion of the components of the CHA_2_DS_2_-VA-score. This model revealed a significant adjusted association between older age and an increased hazard of ATA recurrence (HR = 1.03, 95% CI: 1.01-1.05, *P* = .006). The second model incorporated preoperative clinical variables along with the overall CHA_2_DS_2_-VA-score, where the score itself was significantly associated with an increased hazard of ATA recurrence (HR = 1.23, 95% CI: 1.07-1.41, *P* = .003). The third model combined preoperative variables with periprocedural characteristics, revealing a significant adjusted association of both AF during the postoperative stay (HR = 2.65, 95% CI: 1.77-3.98, *P* < .001) and atrial flutter during the postoperative stay (HR = 2.43, 95% CI: 1.08-5.48, *P* = .032) with ATA recurrences.

### Sex differences in safety and efficacy outcomes

Females undergoing thoracoscopic ablation were older (60.3 vs 57.3, *P* < .001), had more frequent a history of PAF (30.5% vs 15.7%, *P* < .001), and COPD (7.6% vs 2.7%, *P* = .008) compared to male patients (**Table S7**). Female patients presented more frequently with mitral (18.6% vs 10.2%, *P* = .009) and tricuspid valve (13.6% vs 7.7%, *P* = .039) regurgitation (at least moderate) compared to male patients.

There were no overall differences in postoperative complications between females and males; however, female patients more often experienced pulmonary complications, such as pneumonia (2.5% vs 0.5%, *P* = .034) and respiratory failure (3.4% vs 0.5%, *P* = .005) (**Table S8**).

There were no significant differences between females and males regarding long-term freedom from ATA (HR = 1.34, 95% CI: 0.95-1.90, *P* = .095) or treatment failure (HR = 0.94, 95% CI: 0.69-1.30, *P* = .722) (**Figure 2D**).

## Discussion

This study provides a comprehensive real-world evaluation of both isolated thoracoscopic and hybrid thoracoscopic AF ablation, using the bipolar RF clamp. The key findings of this study are: (i) isolated and hybrid thoracoscopic AF ablation with the bipolar RF clamp is a technique with acceptable perioperative morbidity; (ii) freedom from ATA, allowing Class I/III AAD, following thoracoscopic AF ablation was 82.3%, 71.5%, and 52.4% at 1 year, 3 years, and 5 years of follow-up; (iii) freedom from ATA, off Class I/III AAD, following thoracoscopic AF ablation was 71.7%, 60.4%, and 44.2% at 1 year, 3 years, and 5 years of follow-up; (iv) no significant differences were observed in patients with PAF and non-PAF in terms of long-term freedom from ATA, allowing or off Class I/III AAD; (v) while females presented with a more advanced cardiovascular risk profile compared to males, there were no significant sex-based differences in long-term freedom from ATA following ablation.

### Safety and efficacy outcomes

This study is the first and largest retrospective study to present a comprehensive, large-scale, multicentre overview of outcomes for isolated and hybrid thoracoscopic AF ablation using the bipolar RF clamp. It should be emphasized that the reported long-term outcomes predominantly reflect isolated thoracoscopic ablation, as the hybrid cohort represented a smaller component of the registry. In comparing our results on long-term freedom from ATA, Janusauskas et al^3^ previously reported lower ATA freedom off AAD rates of 59%, 41%, and 38% at 1, 3, and 5 years, respectively, in patients undergoing isolated thoracoscopic ablation, compared to the outcomes of the present study. This difference may be attributed to their inclusion of a more complex cohort, including patients with persistent and LSPAF.^4^ A recent meta-analysis by Aerts et al^2^ reported freedom from ATA rates at 1, 3, and 5 years of 71.6%, 55.1%, and 46.8% for thoracoscopic ablation, and 82.0%, 69.9%, and 63.6% for hybrid ablation, incorporating studies that used different energy modalities. However, differences in patient selection, AF subtype distribution, monitoring intensity, and study design preclude firm conclusions regarding comparative efficacy. These findings suggest that the bipolar clamp achieves durable rhythm outcomes comparable to previously published thoracoscopic and hybrid series, although direct comparisons between energy sources cannot be made based on the present study. Although long-term freedom from ATA off AAD declined to 44.2% at 5 years, this should be interpreted in the context of a complex population frequently characterized by persistent AF and advanced atrial remodelling. While these outcomes cannot be considered optimal, they reflect the challenges inherent to surgical treatment of advanced AF substrate and underscore the need for continued refinement of lesion sets and patient selection.

It should also be acknowledged that high-volume centres performing minimally invasive Cox-maze IV procedures have reported higher single-procedure success rates, with low long-term stroke incidence and reduced need for oral anticoagulation.^5^ These results, however, often originate from highly specialized centres with extensive experience and carefully selected patient populations, which may limit generalizability to broader surgical practice.

A subset of patients with PAF did not undergo LAA-management. Unfortunately, data regarding the specific reasons for omission were not uniformly available across centres, although anatomical challenges, procedural preference, or institutional practice variation may have contributed to this finding.

In addition, detailed data regarding the duration and continuity of AAD therapy, particularly amiodarone, were not uniformly available. As some patients may have been monitored while still receiving AAD therapy, this may have influenced reported freedom-from-ATA rates, especially in the early follow-up period. This should be considered when interpreting rhythm outcomes. Although rhythm monitoring protocols were not standardized across centres, most follow-up assessments relied on 24-hour Holter recordings. This approach, while reflective of real-world clinical practice, may have underestimated the true incidence of atrial arrhythmia recurrence. Continuous or extended rhythm monitoring could potentially reveal higher recurrence rates, particularly in patients with asymptomatic episodes. Therefore, the reported efficacy rates should likely be interpreted as conservative estimates of recurrence, and true long-term arrhythmia burden may be higher than captured in the present registry. Therefore, the efficacy outcomes reported in this study should be interpreted with caution, acknowledging that limited monitoring duration represents an inherent methodological challenge.

### Clinical variables associated with freedom from ATA

Interestingly, long-term freedom from ATA was similar between patients with PAF and those with non-PAF. This is notable, given that non-PAF is often linked to more challenging electrophysiological profiles and poorer ablation outcomes. Conversely, the CHA_2_DS_2_-VA-score, and its individual components, emerged as the most valuable preoperative predictor of long-term ATA recurrence.^1^ This finding is consistent with previous reports, where the CHA_2_DS_2_-VASc-score was identified as an essential risk stratification tool for predicting long-term ATA recurrences after catheter ablation.^6^ The association between CHA_2_DS_2_-VA-score and ATA recurrence may be explained by the pathophysiological overlap between thromboembolic risk factors and atrial substrate vulnerability. Components of the score—such as advanced age, hypertension, diabetes, and structural heart disease—are all linked to progressive atrial fibrosis, inflammation, and conduction heterogeneity. These processes facilitate both the initiation and maintenance of ATAs, thereby predisposing to recurrence after ablation. Consequently, the CHA_2_DS_2_-VA-score may serve not only as a marker of stroke risk but also as an indirect measure of atrial disease burden.

### Sex differences

In this cohort, woman represented only 17.4% of the study population and exhibited a higher cardiovascular risk profile compared to men. While these baseline differences may influence long-term outcomes, no significant sex-based differences in freedom from ATA were observed. The limited female representation should, however, be acknowledged as a factor that may affect the generalizability of the results, underscoring the need for more balanced cohorts in future studies.

### Limitations

This study has several limitations. First, the retrospective design of the registry may introduce selection bias, as patient inclusion and procedural choices were based on local clinical practices across different centres. Second, while data were collected from multiple European centres, the variability in follow-up protocols, rhythm monitoring methods, and procedural experience may have contributed to outcome heterogeneity. In addition, data on long-term complications, including stroke and major bleeding events, were not uniformly available across all participating centres, which precluded reliable reporting of these outcomes. Importantly, detailed longitudinal data on oral anticoagulation use, stroke, and major bleeding events were not consistently available. Given that reduction of thromboembolic risk and OAC-related complications represents a major clinical objective of AF surgery, the absence of these outcomes constitutes a significant limitation. Given the retrospective nature of this worldwide outcome database, the use of ECG-based monitoring for outcome assessment was, although suboptimal, at times unavoidable. Moreover, rhythm monitoring was mainly performed using 24-hour Holter recordings, which may have underestimated the true recurrence rate of atrial arrhythmias and represents a major methodological limitation of the study. In addition, short-duration monitoring (24-48 hours) may fail to capture brief, self-terminating ATA episodes, particularly in patients with paroxysmal AF, as highlighted by the 2024 ESC/EACTS guidelines, and could therefore further contribute to underestimation of recurrence rates. Third, the study primarily focused on patients who underwent isolated thoracoscopic ablation, with a smaller cohort receiving hybrid ablation. This imbalance may limit the generalizability of the results to hybrid procedures. Fourth, the reliance on freedom from ATA as a primary end-point, though consistent with current guidelines, does not account for the impact of residual symptoms or quality of life, which are important factors in evaluating procedural success. Fifth, the relatively low proportion of female patients (17.4%) limits the ability to draw robust conclusions regarding sex differences, and future studies with more balanced sex representation are needed to further investigate these findings. Lastly, although long-term outcomes were reported, the median follow-up of 5 years may not fully capture late recurrences of AF, which could emerge beyond this period.

## Conclusion

In this large cohort, both isolated and hybrid thoracoscopic AF ablation using the irrigated bipolar Gemini S Clamp demonstrated acceptable safety and moderate long-term rhythm outcomes in a complex AF population, for patients with both PAF and non-PAF. Key clinical predictors for late ATA recurrence included the CHA_2_DS_2_-VA-score and the presence of early postoperative arrhythmias. Finally, notable sex differences were observed in preoperative cardiovascular risk profiles, although both female and male patients achieved favourable long-term freedom from ATA.

## Supplementary Material

ezag161_Supplementary_Data

## Data Availability

The data underlying this article will be shared on reasonable request to the corresponding author.

## Abbreviations

AAD: Anti-arrhythmic drugs
AF: Atrial fibrillation
ATA: Atrial tachyarrhythmia
KM: Kaplan-Meier
LSPAF: Longstanding persistent AF
Non-PAF: Non-paroxysmal atrial fibrillation
PAF: Paroxysmal atrial fibrillation
PersAF: Persistent AF
PVI: Pulmonary vein isolation
RF: Radiofrequency

## References

[1] Van Gelder IC , Rienstra M, Bunting KV, et al 2024 ESC guidelines for the management of atrial fibrillation developed in collaboration with the European Association for Cardio-Thoracic Surgery (EACTS): developed by the task force for the management of atrial fibrillation of the European Society of Cardiology (ESC), with the special contribution of the European Heart Rhythm Association (EHRA) of the ESC. Endorsed by the European Stroke Organisation (ESO). Eur Heart J 2024;45:3314-3414. 39210723 10.1093/eurheartj/ehae176

[2] Aerts L , Kawczynski MJ, Bidar E, et al Short-and long-term outcomes in thoracoscopic versus hybrid thoracoscopic ablation in patients with atrial fibrillation: a systematic review and reconstructed individual patient data meta-analysis. Europace. 2024;26:euae232. 10.1093/europace/euae102.223 39255332 PMC11448334

[3] Janusauskas V , Puodziukaite L, Maneikiene VV, et al Long-term results of minimally invasive stand-alone bi-atrial surgical ablation with a bipolar ablation device for persistent and longstanding persistent AF: a single-center case series of 91 patients. J Cardiothorac Surg. 2016;11:23. 10.1186/s13019-016-0416-0 26832227 PMC4736089

[4] Maesen B , Weberndörfer V, Bidar E, et al The importance of bipolar bidirectional radiofrequency in surgical AF ablation. Int J Cardiol Heart Vasc. 2020;26:100478. 10.1016/j.ijcha.2020.100478. PMC7046530 32142078

[5] Khiabani AJ , MacGregor RM, Bakir NH, et al The long-term outcomes and durability of the Cox-maze IV procedure for atrial fibrillation. J Thorac Cardiovasc Surg. 2022;163:629-641.e7. 10.1016/j.jtcvs.2020.04.100 32563577 PMC9810144

[6] Kornej J , Hindricks G, Kosiuk J, et al Comparison of CHADS2, R2CHADS2, and CHA2DS2-VASc scores for the prediction of rhythm outcomes after catheter ablation of atrial fibrillation: the Leipzig Heart Center AF ablation registry. Circ Arrhythm Electrophysiol. 2014;7:281-287. 10.1161/CIRCEP.113.001182 24610790

