# Supplementary material for: Safety and Efficacy of Stand-Alone and Hybrid Thoracoscopic Atrial Fibrillation Ablation
Source: Eur J Cardiothorac Surg. 2026 Apr 27;68(5):ezag161. doi: 10.1093/ejcts/ezag161 (PMC13176452; doi:10.1093/ejcts/ezag161)
Supplement: ezag161_Supplementary_Data [file ezag161_supplementary_data.docx]

**Supplementary material**

**Safety and Efficacy of Stand-Alone and Hybrid Thoracoscopic Atrial Fibrillation Ablation.**

*Luca Aerts, MD, Michal J. Kawczynski, MD, Niels J. Verberkmoes, MD, PhD, Thomas Van Brakel, MD, PhD, Justin Luermans, MD, PhD, Samuel Heuts, MD, PhD, Eva Verbeek, PhD, Ricardo Cocchieri, MD, PhD, Sacha P. Salzberg, MD, PhD, Prof, Henri Gruwez, MD, PhD, Herbert Gutermann, MD, Laurent Pison, MD, PhD, Prof, Dmitry Elesin, MD, PhD, Alexander Bogachev-Prokophiev, MD, PhD, Prof, Oleg Shelest, MD, Alexandr Troitskiy, PhD, Robert Khabazov, PhD, Aleksander Zotov, MD, PhD, Bart Maesen, MD, PhD*

Content

**Supplementary Table 1** – Individual center characteristics page 2

**Supplementary Table 2** – Monitoring strategy during the follow-up page 3

**Supplementary Table 3** – Adjusted mixed-effects Cox model for ATA page 4

recurrences

**Supplementary Table 4** – Unadjusted freedom from ATA recurrence page 5

**Supplementary Table 5** – Unadjusted differences for ATA recurrence page 7

**Supplementary Table 6** – Unadjusted differences for Treatment failure page 9

**Supplementary Table 7** – Sex differences for patient characteristics page 10

**Supplementary Table 8** – Sex differences for safety outcomes page 12

**Supplementary Figure 1** - Schematic representation of lesion sets page 13

| **Supplementary Table 1.** Individual center characteristics. | | | | |
| --- | --- | --- | --- | --- |
| **Center** | **Inclusion Date** | **Patients, n (%)** | **Technique** | **Follow up monitoring** |
| Maastricht University Medical Center (MUMC+), Maastricht, The Netherlands | 2019-2022 | 26 (3.83%) | Hybrid AF Ablation | Holter |
| Catharina Hospital, Eindhoven, The Netherlands | 2019-2022 | 65 (9.59%) | Thoracoscopic AF Ablation | Holter |
| Onze Lieve Vrouwe Gasthuis (OLVG), Amsterdam, The Netherlands | 2012-2017 | 112 (16.52%) | Thoracoscopic AF Ablation | ILR |
| Ziekenhuis Zuid Oost Limburg (ZOL), Genk, Belgium | 2019-2023 | 40 (5.90%) | Hybrid AF Ablation | Holter |
| Klinik Hirslanden, Zurich, Switzerland | 2019-2021 | 6  (0.88%) | Thoracoscopic AF Ablation | Holter |
| Schön Klinik Vogtareuth (SKV), Vogtareuth, Germany | 2021-2023 | 24  (3.54%) | Thoracoscopic AF Ablation | Holter |
| Meshalkin National Medical Research Center, Novosibirsk, Russia | 2010-2022 | 298  (43.95%) | Thoracoscopic AF Ablation | Holter |
| Moscow Central Clinical Hospital, Moscow, Russia | 2016-2023 | 107  (15.78%) | Thoracoscopic AF Ablation | Holter |
| AF: atrial fibrillation, ILR: implantable loop recorder | | | | |

| **Supplementary Table 2.** Follow up monitoring. | | | | |
| --- | --- | --- | --- | --- |
| **Follow up** | **Monitoring (%)** | | | |
|  | **24 h Holter** | **48 h Holter** | **7-day Holter** | **ILR** |
| 1 year | 53.39 % | 0.59 % | 1.47 % | 26.25 % |
| 2 year | 54.77 % | 0.56 % | 0.93 % | 31.59 % |
| 3 year | 52.05 % | 0 % | 0.68 % | 35.91 % |
| 4 year | 80.61 % | 0 % | 0 % | 9.89 % |
| 5 year | 88.27 % | 0 % | 0 % | 9.18 % |
| 6 year | 12.5 % | 0 % | 0 % | 0 % |
| *ILR: Implantable Loop Recorder.* | | | | |

| **Supplementary Table 3.** Unadjusted freedom from ATA recurrence. | | | | | | |
| --- | --- | --- | --- | --- | --- | --- |
| **Patient sample** | **1-year** | **2-year** | **3-year** | **4-year** | **5-year** | **P-value (log-rank)** |
| Overall cohort - allowing AAD | 82.3  (79.4-85.3) | 78.6  (75.4-81.8) | 71.5  (67.9-75.4) | 61.8  (57.2-66.7) | 52.4  (47.1-58.3) | *NA* |
| Overall cohort - off AAD^a^ | 71.7  (68.4-75.3) | 66.9  (63.3-70.7) | 60.4  (56.6-64.6) | 51.5  (46.8-56.6) | 44.2  (39.0-50.1) |  |
| Paroxysmal AF-history | 83.8  (77.3-90.7) | 80.2  (73.1-88.1) | 78.8  (71.3-87.1) | 52.5  (23.4-1.00) | *NA* | 0.304 |
| Non-paroxysmal AF-history | 82.0  (78.8-85.3) | 78.2  (74.7-81.9) | 70.2  (66.2-74.5) | 60.9  (56.1-66.0) | 51.6  (46.2-57.6) |  |
| CHA₂DS₂-VA-score 0 | 84.0  (78.0-90.5) | 80.3  (73.6-87.6) | 75.7  (68.2-84.0) | 67.6  (57.3-79.7) | 60.1  (47.6-75.8) | 0.001 |
| CHA₂DS₂-VA-score 1 | 84.4  (80.6-88.4) | 81.6  (77.4-85.9) | 73.2  (68.3-78.6) | 63.6  (57.6-70.2) | 54.3  (47.5-62.0) |  |
| CHA₂DS₂-VA-score 2 | 84.4  (78.2-91.1) | 78.5  (71.4-86.4) | 71.9  (63.8-81.0) | 62.9  (52.7-75.1) | 50.3  (38.0-66.7) |  |
| CHA₂DS₂-VA-score ≥3 | 66.2  (56.3-77.9) | 61.9  (51.4-74.7) | 56.0  (44.4-70.7) | 39.2  (24.6-62.6) | 31.4  (16.5-59.6) |  |
| No arrhythmias during admission | 84.9  (82.0-87.9) | 81.6  (78.4-84.9) | 74.4  (70.7-78.4) | 64.7  (59.9-69.9) | 54.1  (48.4-60.5) | <0.001 |
| Postoperative arrhythmias | 61.1  (50.8-73.5) | 53.9  (43.2-67.3) | 48.1  (37.3-62.2) | 37.8  (26.0-55.0) | 37.8  (26.0-55.0) |  |
| AAD: antiarrhythmic drug, AF: atrial fibrillation, ATA: atrial tachyarrhythmia  ^a^*Off AAD = failure was defined as ATA recurrence or AAD use at any point during FU* | | | | | | |

| **Supplementary Table 4.** Unadjusted differences for any ATA recurrences during the follow-up. | | | | |
| --- | --- | --- | --- | --- |
| **Variable** | **No ATA recurrence**  **(n=465)** | **ATA recurrence**  **(n=213)** | **Hazard Ratio**  **(95% CI)** | **P-value** |
| *Patient characteristics* | | | | |
| Paroxysmal AF-history (%) | 100 (21.5) | 24 (11.3) | 0.87 (0.58-1.31) | 0.509 |
| Prior catheter ablation (%) | 153 (33.3) | 71 (33.5) | 1.35 (1.01-1.79) | **0.043** |
| AF duration, months (IQR) | 44 (24-84) | 45 (24-82) | 1.00 (1.00-1.01) | 0.064 |
| Age, years (SD) | 57.60 (SD: 8.58) | 58.30 (SD: 8.98) | 1.03 (1.01-1.05) | **<0.001** |
| Female sex (%) | 78 (16.8) | 40 (18.8) | 1.34 (0.95-1.90) | 0.095 |
| BSA, m^2^ (SD) | 2.15 (SD: 0.20) | 2.15 (SD: 0.20) | 0.68 (0.33-1.42) | 0.306 |
| BMI, kg/m^2^ (SD) | 29.29 (SD: 4.81) | 29.88 (SD: 4.18) | 1.00 (0.97-1.03) | 0.742 |
| Hypertension (%) | 308 (66.2) | 162 (76.1) | 1.11 (0.81-1.52) | 0.528 |
| Diabetes mellitus (%) | 27 (5.8) | 18 (8.5) | 1.30 (0.80-2.11) | 0.283 |
| Peripheral vascular disease (%) | 23 (4.9) | 12 (5.6) | 1.00 (0.56-1.79) | 0.993 |
| Myocardial infarction (%) | 22 (4.7) | 11 (5.2) | 1.09 (0.59-2.00) | 0.782 |
| History of PCI (%) | 15 (3.2) | 5 (2.3) | 0.87 (0.36-2.12) | 0.763 |
| Stroke (%) | 29 (6.2) | 19 (8.9) | 1.40 (1.10-1.77) | **0.006** |
| Pulmonary embolism (%) | 3 (0.6) | 2 (0.9) | 2.90 (0.71-11.7) | 0.135 |
| COPD (%) | 13 (2.8) | 11 (5.2) | 2.15 (1.17-3.96) | **0.014** |
| Congestive heart failure (%) | 7 (1.5) | 1 (0.5) | 0.50 (0.07-3.59) | 0.492 |
| Kidney dysfunction (%) | 6 (1.3) | 4 (1.9) | 1.73 (0.64-4.65) | 0.281 |
| Sleep apnea (%) | 17 (3.7) | 5 (2.3) | 1.00 (0.41-2.42) | 0.994 |
| Smoking history (%) | 162 (34.8) | 95 (44.6) | 1.36 (1.03-1.81) | **0.028** |
| CHA₂DS₂-VA-score (IQR) | 1 (IQR: 1-2) | 1 (IQR: 1-2) | 1.27 (1.20-1.34) | **<0.001** |
| *Preoperative transthoracic echocardiography* | | | | |
| LVEF, % (SD) | 57.51 (SD: 7.36) | 57.87 (SD: 7.65) | 0.99 (0.97-1.01) | 0.444 |
| LAD, mm (SD) | 47.69 (SD: 7.36) | 50.37 (SD: 8.02) | 1.02 (0.99-1.04) | 0.092 |
| LAV, ml (SD) | 115.26 (SD: 37.21) | 124.87 (SD: 34.12) | 1.00 (0.99-1.00) | 0.496 |
| LAVI, ml/m^2^ (SD) | 53.82 (SD: 16.74) | 58.41 (SD: 15.44) | 1.00 (0.99-1.01) | 0.669 |
| Mitral valve regurgitation (%) | 53 (11.4) | 26 (12.2) | 1.55 (1.02-2.37) | **0.041** |
| Tricuspid valve insufficiency (%) | 37 (7.9) | 22 (10.3) | 1.77 (1.10-2.84) | **0.020** |
| *Procedural characteristics and postoperative course* | | | | |
| Thoracoscopic ablation (%) | 417 (89.7) | 196 (92.0) | 1.58 (0.95-2.62) | 0.077 |
| Hybrid thoracoscopic ablation (%) | 48 (10.3) | 17 (8.0) | 0.63 (0.38-1.05) | 0.077 |
| Surgical PVI + box lesion (%) | 350 (91.4) | 165 (96.5) | 0.87 (0.38-1.98) | 0.739 |
| SVC isolation (%) | 47 (12.3) | 20 (11.8) | 0.48 (0.30-0.78) | **0.003** |
| No LAA management (%) | 18 (3.9) | 6 (2.9) | 0.99 (0.44-2.23) | 0.972 |
| AtriClip (%) | 100 (21.9) | 39 (18.9) | 0.50 (0.35-0.72) | **<0.001** |
| Epicardial stapler device (%) | 338 (74.2) | 161 (78.2) | 1.83 (1.29-2.58) | **<0.001** |
| Procedure time, minutes (SD) | 132.25 (SD: 35.90) | 145.74 (SD: 41.76) | 1.01 (1.00-1.01) | **<0.001** |
| Hospital admission, days (IQR) | 7 (4-8) | 8 (5-9) | 1.00 (0.97-1.04) | 0.896 |
| Sinus rhythm during admission (%) | 426 (91.6) | 175 (82.2) | 0.45 (0.32-0.64) | **<0.001** |
| AF during admission (%) | 22 (4.7) | 27 (12.7) | 2.65 (1.77-3.98) | **<0.001** |
| Atrial flutter during admission (%) | 3 (0.6) | 6 (2.8) | 2.43 (1.08-5.48) | **0.032** |
| Atrial tachycardia during admission (%) | 2 (0.4) | 1 (0.5) | 1.21 (0.17-8.65) | 0.848 |
| Junctional rhythm during admission (%) | 6 (1.3) | 4 (1.9) | 2.38 (0.88-6.41) | 0.087 |
| Other rhythm during admission (%) | 6 (1.3) | 0 (0) | 0.05 (0.00-9.60) | 0.262 |
| AF: atrial fibrillation, BMI: body mass index, BSA: body surface area, COPD: chronic obstructive pulmonary disease, IQR: interquartile range, LAA: left atrial appendage, LAD: left atrial diameter, LAVI: left atrial volume indexed for body surface area, LVEF: left ventricle ejection fraction, NA: not applicable, PAF: paroxysmal atrial fibrillation, PCI: percutaneous coronary intervention, PVI: pulmonary vein isolation, SD: standard deviation, SVC: superior vena cava. | | | | |

| **Supplementary Table 5.** Unadjusted differences for any ATA recurrences or AAD use (treatment failure) during the follow-up. | | | | |
| --- | --- | --- | --- | --- |
| **Variable** | **No treatment failure**  **(n=403)** | **Treatment failure**  **(n=275)** | **Hazard Ratio**  **(95% CI)** | **P-value** |
| *Patient characteristics* | | | | |
| Paroxysmal AF-history (%) | 89 (22.1) | 35 (12.7) | 0.80 (0.56-1.15) | 0.230 |
| Prior catheter ablation (%) | 134 (33.6) | 90 (33.0) | 1.23 (0.96-1.59) | 0.105 |
| AF duration, months (IQR) | 45 (24-84) | 42 (24-84) | 1.00 (1.00-1.00) | 0.074 |
| Age, years (SD) | 57.54 (SD: 8.54) | 58.23 (SD: 8.95) | 1.02 (1.01-1.04) | **0.003** |
| Female sex (%) | 72 (17.9) | 46 (16.7) | 0.94 (0.69-1.30) | 0.722 |
| BSA, m^2^ (SD) | 2.15 (SD: 0.20) | 2.15 (SD: 0.20) | 0.94 (0.50-1.78) | 0.850 |
| BMI, kg/m^2^ (SD) | 29.30 (SD: 4.92) | 29.72 (SD: 4.15) | 0.99 (0.97-1.02) | 0.655 |
| Hypertension (%) | 266 (66.0) | 204 (74.2) | 1.08 (0.82-1.41) | 0.592 |
| Diabetes mellitus (%) | 26 (6.5) | 19 (6.9) | 1.03 (0.65-1.64) | 0.909 |
| Peripheral vascular disease (%) | 22 (5.5) | 13 (4.7) | 0.81 (0.47-1.42) | 0.464 |
| Myocardial infarction (%) | 19 (4.7) | 14 (5.1) | 1.07 (0.63-1.84) | 0.796 |
| History of PCI (%) | 14 (3.5) | 6 (2.2) | 0.72 (0.32-1.63) | 0.433 |
| Stroke (%) | 28 (6.9) | 20 (7.3) | 1.17 (0.93-1.47) | 0.184 |
| Pulmonary embolism (%) | 3 (0.7) | 2 (0.7) | 2.10 (0.52-8.44) | 0.297 |
| COPD (%) | 12 (3.0) | 12 (4.4) | 1.61 (0.90-2.88) | 0.106 |
| Congestive heart failure (%) | 6 (1.5) | 2 (0.7) | 0.68 (0.17-2.75) | 0.591 |
| Kidney dysfunction (%) | 5 (1.2) | 5 (1.8) | 1.57 (0.65-3.81) | 0.318 |
| Sleep apnea (%) | 17 (4.2) | 5 (1.8) | 0.68 (0.28-1.64) | 0.385 |
| Smoking history (%) | 143 (35.5) | 115 (41.8) | 1.27 (1.00-1.62) | 0.050 |
| CHA₂DS₂-VA-score (IQR) | 1 (IQR: 1-2) | 1 (IQR: 1-2) | 1.15 (1.09-1.21) | **<0.001** |
| *Preoperative transthoracic echocardiography* | | | | |
| LVEF, % (SD) | 57.36 (SD: 7.59) | 58.00 (SD: 7.24) | 1.00 (0.98-1.02) | 0.913 |
| LAD, mm (SD) | 47.78 (SD: 7.37) | 49.59 (SD: 7.96) | 1.01 (0.99-1.03) | 0.279 |
| LAV, ml (SD) | 114.84 (SD: 37.47) | 123.33 (SD: 34.55) | 1.00 (0.99-1.00) | 0.872 |
| LAVI, ml/m^2^ (SD) | 53.78 (SD: 16.92) | 57.47 (SD: 15.57) | 1.00 (0.99-1.01) | 0.762 |
| Mitral valve regurgitation (%) | 45 (11.2) | 34 (12.4) | 1.36 (0.94-1.98) | 0.082 |
| Tricuspid valve insufficiency (%) | 33 (8.2) | 26 (9.5) | 1.46 (0.95-2.23) | 0.084 |
| *Procedural characteristics and postoperative course* | | | | |
| Thoracoscopic ablation (%) | 358 (88.8) | 255 (92.7) | 1.18 (0.75-1.87) | 0.482 |
| Hybrid thoracoscopic ablation (%) | 45 (11.2) | 20 (7.3) | 0.85 (0.54-1.34) | 0.482 |
| Surgical PVI + box lesion (%) | 307 (90.6) | 208 (96.7) | 1.15 (0.54-2.46) | 0.714 |
| SVC isolation (%) | 44 (13.0) | 23 (10.7) | 0.65 (0.42-1.01) | 0.053 |
| No LAA management (%) | 16 (4.1) | 8 (3.0) | 0.92 (0.45-1.86) | 0.814 |
| AtriClip (%) | 87 (22.1) | 52 (19.4) | 0.59 (0.43-0.81) | **<0.001** |
| Epicardial stapler device (%) | 291 (73.9) | 208 (77.6) | 1.55 (1.15-2.08) | **0.004** |
| Procedure time, minutes (SD) | 131.17 (SD: 35.07) | 144.36 (SD: 41.51) | 1.01 (1.00-1.01) | **<0.001** |
| Hospital admission, days (IQR) | 7 (4-8) | 8 (5-9) | 1.03 (0.99-1.06) | 0.093 |
| Sinus rhythm during admission (%) | 372 (92.3) | 229 (83.3) | 0.49 (0.36-0.67) | **<0.001** |
| AF during admission (%) | 17 (4.2) | 32 (11.6) | 2.35 (1.63-3.41) | **<0.001** |
| Atrial flutter during admission (%) | 2 (0.5) | 7 (2.5) | 2.55 (1.20-5.40) | **0.015** |
| Atrial tachycardia during admission (%) | 1 (0.2) | 2 (0.7) | 1.76 (0.44-7.09) | 0.424 |
| Junctional rhythm during admission (%) | 6 (1.5) | 4 (1.5) | 1.61 (0.60-4.33) | 0.343 |
| Other rhythm during admission (%) | 5 (1.2) | 1 (0.4) | 0.27 (0.04-1.93) | 0.192 |
| AF: atrial fibrillation, BMI: body mass index, BSA: body surface area, COPD: chronic obstructive pulmonary disease, IQR: interquartile range, LAA: left atrial appendage, LAD: left atrial diameter, LAVI: left atrial volume indexed for body surface area, LVEF: left ventricle ejection fraction, NA: not applicable, PAF: paroxysmal atrial fibrillation, PCI: percutaneous coronary intervention, PVI: pulmonary vein isolation, SD: standard deviation, SVC: superior vena cava. | | | | |

| **Supplementary table 6.** Adjusted mixed-effects Cox model for ATA recurrence. | | | | | | |
| --- | --- | --- | --- | --- | --- | --- |
| Variable | *Model 1* | | *Model 2* | | *Model 3* | |
|  | Hazard ratio  (95% CI) | *P*-value | Hazard ratio  (95% CI) | *P*-value | Hazard ratio  (95% CI) | *P*-value |
| Age, per 1 year | 1.03 (1.01-1.05) | 0.006 | - | - | - | - |
| Female sex | 1.28 (0.89-1.85) | 0.19 | - | - | - | - |
| Stroke history | 1.50 (0.88-2.55) | 0.13 | - | - | - | - |
| CHA₂DS₂-VA-score | - | - | 1.23 (1.07-1.41) | 0.003 | 1.24 (1.08-1.42) | <0.001 |
| Prior catheter ablation | 1.25 (0.91-1.71) | 0.16 | 1.18 (0.87-1.61) | 0.29 | 1.19 (0.87-1.63) | 0.27 |
| AF-history duration, per 1 month | 1.00 (1.00-1.00) | 0.99 | 1.00 (1.00-1.00) | 0.80 | 1.00 (1.00-1.00) | 0.95 |
| Paroxysmal AF-history | 0.73 (0.44-1.21) | 0.22 | 0.76 (0.46-1.25) | 0.27 | 0.74 (0.45-1.17) | 0.24 |
| COPD history | 1.29 (0.64-2.59) | 0.48 | 1.37 (0.68-2.75) | 0.38 | 1.39 (0.69-2.80) | 0.35 |
| Sinus rhythm during admission | - | - | - | - | 0.46 (0.30-0.68) | <0.001 |
| Hybrid thoracoscopic ablation | - | - | - | - | 0.94 (0.40-2.22) | 0.89 |
| ILR monitoring for AF recurrence | - | - | - | - | 0.93 (0.61-1.45) | 0.72 |
| *Schoenfeld residuals* | - | 0.555 | - | 0.394 | - | 0.157 |
| AF: atrial fibrillation, CI: confidence interval, COPD: chronic obstructive pulmonary disease, HR: hazard ratio. | | | | | | |

| **Supplementary Table 7.** Baseline patient characteristics stratified by sex | | | | |
| --- | --- | --- | --- | --- |
| **Variable** | **Overall cohort (n=678)** | **Females**  **(n=118)** | **Males**  **(n=560)** | **P-value** |
| *Patient characteristics* | | | | |
| Preoperative rhythm history (%) | 678 (100) | 118 (100) | 560 (100) | **<0.001** |
| Paroxysmal AF (%) | 124 (18.3) | 36 (30.5) | 88 (15.7) | **<0.001** |
| Persistent AF (%) | 102 (15.0) | 17 (14.4) | 85 (15.2) | 0.831 |
| Longstanding persistent AF (%) | 452 (66.7) | 65 (55.1) | 387 (69.1) | **0.003** |
| Prior catheter ablation (%) | 224 (33.3) | 34 (29.1) | 190 (34.2) | 0.281 |
| AF duration, months (IQR) | 45 (24-84) | 36 (24-88) | 45 (24-81) | 0.672 |
| Age, years (SD) | 57.82 (SD: 8.71) | 60.33 (SD: 8.68) | 57.29±8.63 | **<0.001** |
| BSA, m^2^ (SD) | 2.15 (SD: 0.20) | 1.97(SD: 0.19) | 2.19±0.18 | **<0.001** |
| BMI, kg/m^2^ (SD) | 29.48 (SD: 4.63) | 30.09 (SD: 5.40) | 29.35±4.44 | 0.113 |
| Hypertension (%) | 470 (69.3) | 76 (64.4) | 394 (70.4) | 0.203 |
| Diabetes mellitus (%) | 45 (6.6) | 10 (8.5) | 35 (6.3) | 0.378 |
| Peripheral vascular disease (%) | 35 (5.2) | 5 (4.2) | 30 (5.4) | 0.617 |
| Myocardial infarction (%) | 33 (4.9) | 4 (3.4) | 29 (5.2) | 0.412 |
| History of PCI (%) | 21 (3.1) | 2 (1.7) | 19 (3.2) | 0.375 |
| Stroke (%) | 48 (7.1) | 11 (9.3) | 37 (6.6) | 0.296 |
| Pulmonary embolism (%) | 5 (0.7) | 1 (0.8) | 4 (0.7) | 0.878 |
| COPD (%) | 24 (3.5) | 9 (7.6) | 15 (2.7) | **0.008** |
| Congestive heart failure (%) | 8 (1.2) | 2 (1.7) | 6 (1.1) | 0.569 |
| Kidney dysfunction (%) | 10 (1.5) | 3 (2.6) | 7 (1.3) | 0.285 |
| Sleep apnea (%) | 22 (3.2) | 2 (1.7) | 20 (3.6) | 0.296 |
| Smoking history (%) | 258 (38.1) | 28 (23.7) | 230 (41.1) | **<0.001** |
| CHA₂DS₂-VASc-score (IQR) | 1 (1-2) | 2 (2-3) | 1 (1-2) | **<0.001** |
| CHA₂DS₂-VA-score (IQR) | 1 (IQR: 1-2) | 1 (IQR: 1-2) | 1 (IQR: 1-2) | **0.002** |
| *Preoperative medication* | | | | |
| Antiarrhythmic drugs (%) | 644 (100) | 108 (100) | 536 (100) | 0.235 |
| No antiarrhythmic drugs | 160 (24.8) | 32 (29.6) | 128 (23.9) | 0.207 |
| Flecainide | 52 (8.1) | 11 (10.2) | 41 (7.6) | 0.378 |
| Procainamide | 3 (0.5) | 1 (0.9) | 2 (0.4) | 0.442 |
| Sotalol | 87 (13.5) | 18 (16.7) | 69 (12.9) | 0.293 |
| Amiodarone | 337 (52.3) | 46 (42.6) | 291 (54.3) | **0.026** |
| Other | 5 (0.8) | 0 (0) | 5 (0.9) | 0.314 |
| Oral anticoagulation (%) | 636 (93.8) | 108 (91.5) | 528 (94.3) | 0.258 |
| *Preoperative transthoracic echocardiography* | |  |  |  |
| LVEF, % (SD) | 57.62 (SD: 7.45) | 57.67 (SD: 8.35) | 57.61±7.25 | 0.941 |
| LAD, mm (SD) | 48.57 (SD: 7.68) | 45.91 (SD: 8.02) | 49.02±7.53 | **0.003** |
| LAV, ml (SD) | 118.47 (SD: 36.46) | 102.63 (SD: 36.78) | 121.54±35.63 | **<0.001** |
| LAVI, ml/m^2^ (SD) | 55.36 (SD: 16.44) | 52.48 (SD: 17.90) | 55.92±16.10 | 0.084 |
| Mitral valve regurgitation (%)^a^ | 79 (11.7) | 22 (18.6) | 57 (10.2) | **0.009** |
| Tricuspid valve insufficiency (%)^b^ | 59 (8.7) | 16 (13.6) | 43 (7.7) | **0.039** |
| AF: atrial fibrillation, BMI: body mass index, BSA: body surface area, COPD: chronic obstructive pulmonary disease, LAD: left atrial diameter, LAVI: left atrial volume indexed for body surface area, LVEF: left ventricle ejection fraction, PAF: paroxysmal atrial fibrillation, PCI: percutaneous coronary intervention, SD: standard deviation.  ^a^Defined as moderate or more severe regurgitation  ^b^Defined as moderate or more severe regurgitation | | | | |

| **Supplementary Table 8.** Sex differences in postoperative course and early outcomes | | | | |
| --- | --- | --- | --- | --- |
| **Variable** | **Overall cohort (n=678)** | **Females**  **(n=118)** | **Males**  **(n=560)** | **P-value** |
| *Patient characteristics* | | | | |
| ICU admission, days (IQR) | 1 (1-1) | 1 (1-1) | 1 (1-1) | 0.324 |
| Hospital admission, days (IQR) | 7 (4-9) | 6 (4-8) | 7 (4-9) | 0.060 |
| Rhythm during admission (%) | 678 (100) | 118 (100) | 560 (100) | 0.874 |
| Sinus rhythm (%) | 601 (88.6) | 103 (87.3) | 498 (88.9) | 0.610 |
| AF (%) | 49 (7.2) | 11 (9.3) | 38 (6.8) | 0.334 |
| Atrial flutter (%) | 9 (1.3) | 2 (1.7) | 7 (1.3) | 0.701 |
| Atrial tachycardia (%) | 3 (0.4) | 0 (0) | 3 (0.5) | 0.426 |
| Junctional rhythm (%) | 10 (1.4) | 1 (0.8) | 9 (1.7) | 0.533 |
| Other (%) | 6 (0.9) | 1 (0.8) | 5 (0.9) | 0.962 |
| In-hospital mortality (%) | 1 (0.1) | 0 (0) | 1 (0.2) | 0.644 |
| Postoperative complications (%) | 60 (8.8) | 13 (11.0) | 47 (8.4) | 0.362 |
| Pneumothorax requiring drainage (%) | 10 (1.5) | 2 (1.7) | 8 (1.4) | 0.827 |
| Pleural effusion requiring drainage (%) | 12 (1.8) | 1 (0.8) | 11 (2.0) | 0.403 |
| Hemothorax requiring drainage (%) | 3 (0.4) | 0 (0) | 3 (0.5) | 0.426 |
| Respiratory failure (%) | 7 (1.0) | 4 (3.4) | 3 (0.5) | **0.005** |
| Pneumonia (%) | 6 (0.9) | 3 (2.5) | 3 (0.5) | **0.034** |
| Pericarditis (%) | 7 (1.0) | 0 (0) | 7 (1.3) | 0.222 |
| Stroke (%) | 4 (0.6) | 1 (0.8) | 3 (0.5) | 0.688 |
| Permanent PM implantation (%) | 7 (1.0) | 1 (0.8) | 6 (1.1) | 0.827 |
| Phrenic nerve palsy (%) | 1 (0.1) | 1 (0.8) | 0 (0) | 0.174 |
| Ventricular fibrillation (%) | 1 (0.1) | 0 (0) | 1 (0.2) | 0.646 |
| Myocardial infarction (%) | 2 (0.3) | 0 (0) | 2 (0.4) | 0.516 |
| AF: atrial fibrillation, ICU: intensive care unit, IQR: interquartile range, PM: pacemaker | | | | |

**Supplementary Figure 1** Schematic representation of lesion sets

**
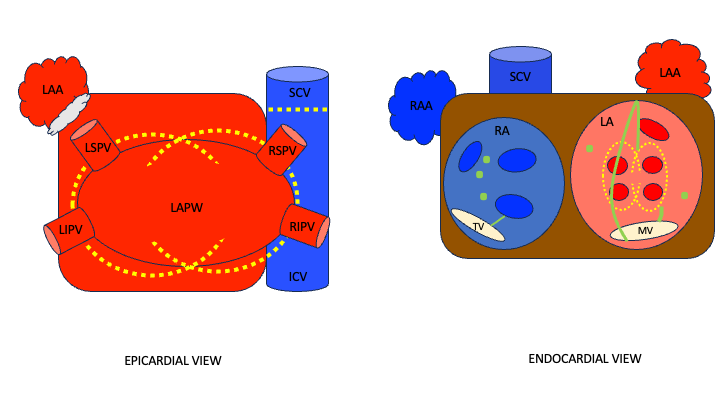
**

Schematic overview of the lesion sets from an epicardial and endocardial perspective. Yellow dashed lines depict the epicardial left atrial posterior wall (LAPW) box lesion. Green dots in the right atrium (RA) and left atrium (LA) represent focal endocardial ablations. The green line between the orifice of the inferior caval vein (ICV) and the tricuspid valve (TV) represents the cavotricuspid isthmus. The green lines in the LA between the mitral valve and the epicardial LAPW lesion represent the lateral and anterior mitral isthmus line. The left atrial appendage (LAA) is clipped in the epicardial view.

*Legend: ICV: inferior caval vein; LA: left atrium; LAA: left atrial appendage; LAPW: left atrial posterior wall; LIPV: left inferior pulmonary vein; LSPV: left superior pulmonary vein; MV: mitral valve; RA: right atrium; RAA: right atrial appendage; RIPV: right inferior pulmonary vein; RSPV: right superior pulmonary vein; SCV: superior caval vein; TV: tricuspid valve.*
